# *IL28B* gene polymorphisms in mono- and HIV-coinfected chronic hepatitis C patients

**DOI:** 10.3389/fmicb.2015.00153

**Published:** 2015-03-04

**Authors:** Bruna C. Bertol, Simone Moreira, Raquel F. L. Garcia, Leslie E. Ferreira, Guilherme Debortoli, Mauro de Souza Leite Pinho, Marcia Amendola-Pires, Alessandra M. de Almeida Maciel, Carlos E. Brandço-Mello, Paulo H. C. de França

**Affiliations:** ^1^Laboratory of Molecular Biology, Department of Medicine, Universidade da Região de JoinvilleJoinville, Brazil; ^2^Hospital Municipal São José, JoinvilleBrazil; ^3^Hospital Universitário Gaffrée Guinle – Universidade Federal do Estado do Rio de Janeiro, Rio de JaneiroBrazil

**Keywords:** hepatitis C, HIV, HCV–HIV coinfection, *IL28B* gene, single nucleotide polymorphisms (SNPs)

## Abstract

**Introduction:** Single-nucleotide polymorphisms (SNPs) associated with hepatitis C virus (HCV) clearance were identified near the *IL28B* gene. Coinfection by the human immunodeficiency virus (HIV) influences the course of HCV contributing to liver damage. Nevertheless, little is known about the relationship between these SNPs and HCV/HIV coinfection. Our aim was to estimate the frequencies of the allelic and genotypic variants of the *IL28B* polymorphisms rs12979860 (*C*/*T*) and rs8099917 (*T*/*G*) and their possible association with the establishment of HCV infection.

**Methodology:** A total of 199 non-infected controls and 230 patients with chronic hepatitis C, including 53 coinfected with HIV, participated in the study. Genotyping consisted of polymerase chain reaction and subsequent analysis of the restriction patterns resulting from exposure to endonucleases.

**Results:** Among the controls with established results, 47.4% (90/190) exhibited the rs12979860 *CC* genotype, 43.7 *CT*, and 8.9% *TT*, whereas 29.1% (66/227), 51.5%, and 19.4% of the patients exhibited the *CC*, *CT*, and *TT* genotypes, respectively. With respect to rs8099917, 66.8% (133/199) of the controls exhibited the *TT* genotype, 31.2% *TG*, and 2.0% *GG*, whereas 56.1% (129/230), 40.9%, and 3.0% of the patients exhibited the *TT*, *TG*, and *GG* genotypes, respectively.

**Conclusion:** The frequencies of the rs12979860 *C* allele and *CC* genotype and of the rs8099917 *T* allele and *TT* genotype were significantly higher among controls compared with patients, thus confirming the suggested protective effect against HCV infection. No significant difference was observed in the genotype and allelic distributions between the mono- and coinfected patients.

## INTRODUCTION

Hepatitis C is caused by the hepatitis C virus (HCV), an enveloped, single-stranded RNA virus that belongs to the family *Flaviviridae* (World Health Organization [WHO], 2014). Approximately 70–80% of HCV infections become chronic; only a small fraction of infected individuals exhibit spontaneous viral clearance. The patients with active persistent infection are at high risk of developing cirrhosis and hepatocellular carcinoma (Seeff, 2002). The most cost effective means of controlling infectious disease is through vaccination, however, a prophylactic or therapeutic vaccine for HCV is not available yet (Drummer, 2014). Until 2011, the standard treatment of chronic hepatitis C consists of a combination of pegylated interferon-α (PegIFN-α-2a/2b) and ribavirin (RBV). However, the efficacy of the standard treatment was limited, as only 45–55% of the patients infected by HCV genotype 1 achieved the sustained virological response (SVR)(García et al., 2001). After 2011, direct antiviral agents (DAAs) for the treatment of patients with HCV genotype 1 became available in many countries, namely boceprevir (BOC) and telaprevir (TVR), used in combination with PegIFN and RBV, called triple-therapy, to improve cure rates (Bacon et al., 2011; Jacobson et al., 2011; Poordad et al., 2011).

Every year, 3–4 millions of people worldwide are infected by the HCV, and the number of individuals with chronic infection is estimated to be approximately 130–150 million (World Health Organization [WHO], 2014). In Brazil, the estimated number of individuals with hepatitis C is two million (Ciorlia and Zanetta, 2007). The prevalence of the human immunodeficiency virus (HIV) infection among HCV-infected individuals is high, largely because the major viral transmission pathways are the same for these infections. For instance, in US and Western Europe, the prevalence of the coinfection is as elevated as 72% to 95% among injection drug users (Operskalski and Kovacs, 2011). Coinfection is characterized by the impact of HIV on the natural course of HCV infection and vice-versa. HIV accelerates the progression of liver disease in HCV-infected individuals, thus increasing the risk of cirrhosis and viremia rates while reducing the odds of attaining an SVR to interferon-based treatment (Martino et al., 2001; Silva and Barone, 2006).

Ge et al. (2009), Tanaka et al. (2009), and Thomas et al. (2009) were the first to identify Single Nucleotide Polymorphisms (SNPs) near the *IL28B* gene on chromosome 19 that exhibit strong associations with HCV clearance. The *CC* rs12979860 and *TT* rs8099917 genotypes are associated with a significant increase in the rate of spontaneous resolution of HCV infection as well as increased odds of attaining SVR in mono- and coinfected patients (Aparicio et al., 2010; Rállon et al., 2010; Lunge et al., 2012). In addition, Ge et al. (2009) determined that the *C* allele frequency of the rs12979860 was significantly reduced in a group of patients with chronic hepatitis C compared to a matched control group (*p* < 2.5 × 10^-6^).

Despite the evidence of the relevance of polymorphisms rs12979860 and rs8099917 in hepatitis C, there have been few studies on their prevalence in different groups of individuals. Additionally, few studies have investigated the influence of SNPs on the establishment of HCV infection in individuals also infected with HIV. Thus, the aim of the study was to estimate the frequencies of the allelic and genotypic variants of the *IL28B* polymorphisms rs12979860 (*C/T*) and rs8099917 (*T/G*) and their possible association with the establishment of HCV infection in HCV mono-infected and HCV/HIV coinfected patients.

## MATERIALS AND METHODS

### STUDY POPULATION AND DNA EXTRACTION

Individuals with serological evidence of non-exposure to HCV and HIV were recruited among blood donors and composed the control group. The HCV mono-infected and the HCV/HIV-1 coinfected patients were consecutively recruited at Hospital Universitário Gaffrée & Guinle (Rio de Janeiro, RJ, Brazil), reflecting the local casuistic.

The peripheral blood collected by finger puncture from mono- and coinfected patients was collected using filter paper (FTA Elute Micro Card®;, Whatman, Kent, UK). To extract the genomic DNA, 3-mm diameter disks were removed from the cards and processed according to the manufacturer’s instructions. SNP-based genotyping for rs12979860 was performed in 190 non-infected individuals and 227 patients, 52 of whom were coinfected. In addition, SNP-based genotyping for rs8099917 was performed in 199 non-infected individuals and 230 patients, 53 of whom were coinfected. The study was approved by the Research Ethics Committee of Universidade da Região de Joinville (UNIVILLE; protocol 136/10). Informed consent was obtained from each participant before blood sampling.

### GENETIC ANALYSIS

The rs12979860 and rs8099917 SNP variants were identified as developed by Moreira et al. (2012). First, polymerase chain reaction (PCR) was performed in the LGC XP thermocycler (BIOER Technology Co., Tokio, Japan) using specific oligonucleotides to obtain the genetic segments containing the SNPs rs12979860 and rs8099917. The *amplicons* containing SNPs rs12979860 and rs8099917 were 694 and 496 base pairs (bp), respectively. The *amplicons* were subjected to Restriction Fragment Length Polymorphism (RFLP) analysis using the *BsrD*I and *Hpy*166II endonucleases (New England Biolabs, Beverly, MA, USA) for rs12979860 and rs8099917, respectively. Finally, the products of enzymatic digestion were subjected to agarose gel electrophoresis. The results were visualized using MiniBis-Pro documentation system (DNR Bio-Imaging Systems Ltd., Jerusalem, Israel). Thus, the genotype of each investigated individual was established based on the analysis of the electrophoretic profile: *CC* homozygous (two fragments of 509 and 185 bp), *TT* homozygous (509, 155, and 30 bp), or *CT* heterozygous (509, 185, 155, and 30 bp) for rs12979860 and *TT* homozygous (496 bp), *GG* homozygous (272 and 224 bp), or *GT* heterozygous (496, 272, and 224 bp) for rs8099917. PCR and RFLP analyses were performed independently for each investigated polymorphism, thus representing two separate procedures. All of the analyses were performed at the Laboratory of Molecular Biology of UNIVILLE.

### TREATMENT OF DATA AND STATISTICAL ANALYSIS

The adherence of the identified genotype frequencies to the Hardy–Weinberg theoretical proportions, the SNP linkage disequilibrium analysis, and the allelic and genotype frequencies of each polymorphism in cases and controls were estimated using the software GENEPOP version 3.4 (available at http://www.biomed.curtin.edu.au/genepop) (Raymond and Rousset, 1995). Linkage disequilibrium and Hardy–Weinberg equilibrium were defined by *p*-values lower and higher than 0.05, respectively. The association of the genotypes with the susceptibility to HCV infection was established based on 2 × 2 contingency tables by means of the Odds Ratio (OR) with 95% Confidence Intervals (95% CI), considering *p*-values lower than 0.05 to be significant (Woolf, 1955). This test was performed using the Epimax Table Calculator software (Health Decision Strategies [HDS], 2013). The HCV mono- and coinfected patients were subjected to both joint and separate analyses to assess the influence of HIV on the results.

## RESULTS

### CHARACTERISTICS OF THE STUDY POPULATION

HIV-infected patients were receiving successful antiretroviral therapy and had an undetectable viral load at the time of blood sampling. The demographic and virological characteristics of the patients chronically mono-infected with HCV, those coinfected with HIV-1, and the non-infected individuals are described in **Table 1**.

**Table 1:** Demographic and virological characteristics of study population.

	Controls	Patients (total)	HCV patients	HCV/HIV-1 patients
Mean age (years ± SD)	34.3 ± 10.61	55.2 ± 9.88	56.8 ± 9.60	50 ± 9.13
**Gender** Female Male	68/199 (34.2%) 131/199 (65.8%)	91/226 (40.3%) 135/226 (59.7%)	81/173 (46.8%) 192/173 (53.2%)	10/53 (18.9%) 43/53 (81.1%)
**HCV genotype**
1 Others	– –	210/218 (96.3%) 8/218 (3.7%)	157/165 (95.1%) 8/165 (4.9%)	53/53 (100%) –

*SD, standard deviation*.

### POLYMORPHISMS rs12979860 AND rs8099917 And Linkage Disequilibrium

The analysis of the probability of segregation between polymorphic sites was performed by means of the linkage disequilibrium test. The investigated SNPs (rs12979860 and rs8099917) proved to be ligated in all of the populations compared in the present study: non-infected individuals vs. coinfected patients; non-infected individuals vs. mono-infected patients; and coinfected vs. mono-infected patients. All of these comparisons exhibited *p*-values lower than 0.05.

### POLYMORPHISM rs12979860 IN NON-INFECTED INDIVIDUALS AND PATIENTS

The distribution frequency of the SNP rs12979860 among the non-infected individuals and patients complied with Hardy–Weinberg equilibrium. **Table 2** describes the genotypic and allelic frequencies exhibited by the non-infected individuals and by the mono- and coinfected patients with chronic hepatitis C.

**Table 2:** Allelic and genotypic frequencies and association analysis – rs12979860.

	Controls (*n* = 190)	Patients (total) (*n* = 227)	Comparison	OR (CI 95%)	*p*
**Genotype**
*CC*	90 (47.4%)	66 (29.1%)	–	–	–
*CT*	83 (43.7%)	117 (51.5%)	TT+CT vs. CC	2.195 (1.437–3.357)	<0.001*
*TT*	17 (8.9%)	44 (19.4%)	TT vs. CT+CC	2.447 (1.300–4.648)	0.005*
*p*	–	<0.001^A^*	–	–	–
**Allele**
* C*	263 (69.2%)	249 (54.9%)	–	–	–
*T*	117 (30.8%)	205 (45.1%)	T vs. C	1.851 (1.377–2.488)	<0.001*
*p*	–	<0.001^B^*	–	–	–

	**Controls (***n*** = 190)**	**HCV patients (***n*** = 175)**	**Comparison**	**OR (CI 95%)**	*****p*****

**Genotype**



*CC*	90 (47.4%)	54 (30.9%)	–	–	–
*CT*	83 (43.7%)	84 (48.0%)	TT+CT vs. CC	2.017 (1.284–3.170)	0.002*
*TT*	17 (8.9%)	37 (21.1%)	TT vs. CT+CC	2.728 (1.417–5.296)	0.002*
*p*	–	<0.001^C^*	–	–	–
**Allele**
* C*	263 (69.2%)	192 (54.9%)	–	–	–
*T*	117 (30.8%)	158 (45.1%)	T vs. C	1.850 (1.351–2.534)	<0.001*
*p*	–	<0.001^D^*	–	–	–

	**Controls (***n*** = 190)**	**HCV/HIV-1 patients (***n*** = 52)**	**Comparison**	**OR (CI 95%)**	*****p*****

**Genotype**



*CC*	90 (47.4%)	12 (23.5%)	–	–	–
*CT*	83 (43.7%)	33 (62.8%)	TT+CT vs. CC	3.000 (1.413–6.469)	0.003*
*TT*	17 (8.9%)	7 (13.7%)	TT vs. CT+CC	1.583 (0.556–4.374)	0.482
*p*	–	0.007^E^*	–	–	–
**Allele**
* C*	263 (69.2%)	57 (54.8%)	–	–	-
*T*	117 (30.8%)	47 (45.2%)	T vs. C	1.854 (1.162–2.957)	0.009*
*p*	–	0.009^F^*	–	–	–

	**HCV patients (***n*** = 175)**	**HCV/HIV-1 patients (***n*** = 52)**	**Comparison**	**OR (CI 95%)**	*****p*****

**Genotype**



*CC*	54 (30.9%)	12 (23.5%)	–	–	–
*CT*	84 (48.0%)	33 (62.8%)	TT+CT vs. CC	1.488 (0.688–3.263)	0.363
*TT*	37 (21.1%)	7 (13.7%)	TT vs. CT+CC	0.580 (0.219–1.479)	0.303
*p*	–	1.000^G^	–	–	–
**Allele**
* C*	192 (54.9%)	57 (54.8%)	–	–	–
*T*	158 (45.1%)	47 (45.2%)	T vs. C	1.002 (0.630–1.592)	1.000
*p*	–	1.000^H^	–	–	–

^A,B^Controls vs. Patients (total); ^C,D^Controls vs. HCV Patients; ^E,F^Controls v*s.* HCV/HIV-1 Patients; ^G,H^HCV Patients vs; HCV/HIV-1 Patients; OR, Odds ratio; CI, Confidence intervals. *Statistical significance.

Significant differences were observed in the genotype frequencies; the *CC* genotype was more prevalent in non-infected individuals (47.4%, *p* < 0.001) compared to the whole group (mono- and coinfected) of patients (29.1%). In addition, the *C* allele was observed more frequently in non-infected individuals (69.2%, *p* < 0.001) than in the patients group (54.9%). An analysis of the relative risk of this SNP relative to the susceptibility to viral infection revealed an OR of 2.447 (*p* = 0.005) for the *TT* genotype alone and an OR of 2.195 (*p* < 0.001) for the genotypes containing at least one *T* allele (*TT* and *CT*) in the chronic hepatitis C population (mono- and coinfected patients taken together). Similar result was obtained in the analysis of the relative risk of *T* allele (OR = 1.851, *p* < 0.001).

Significant differences were also observed in the distribution of alleles and genotypes in non-infected individuals compared to the mono- and coinfected patients when analyzed separately (**Table 2**). The *CC* genotype was more frequent in non-infected individuals (47.4%) compared to mono-infected (30.9%, *p* < 0.001) and coinfected (23.5%, *p* = 0.007) patients. Additionally, the *C* allele was more frequently observed in non-infected patients (69.2%) compared with both groups of patients (mono-infected – 54.9%, *p* < 0.001; coinfected – 54.8%, *p* = 0.009). An analysis of the relative risk of this SNP in the group of mono-infected patients indicated an OR of 2.728 (*p* = 0.002) for the *TT* genotype, an OR of 2.017 (*p* = 0.002) for genotypes containing at least one allele *T* (*TT* and *CT*), and an OR of 1.852 (*p* < 0.001) for the *T* allele. In the group of coinfected patients, the *TT* genotype, *TT*+*CT* genotypes, and *T* allele exhibited ORs of 1.583 (*p* = 0.482), 3.000 (*p* = 0.003), and 1.846 (*p* = 0.009), respectively.

No significant difference was observed in the distributions of the *CC* genotype (30.9% vs. 23.5%, *p* = 1.000) and the *C* allele (54.9% vs. 54.8%, *p* = 1.000) between the groups of mono- and coinfected patients, respectively. Neither risk factor analysis indicated any relevant risk for either genotypes (*TT* vs. *CT*+*CC*, OR = 0.58, *p* = 0.303; *TT*+*CT* vs. *CC*, OR = 1.488, *p* = 0.363) or alleles (OR = 1.002, *p* = 1.000).

### POLYMORPHISM rs8099917 IN NON-INFECTED INDIVIDUALS AND PATIENTS

The distribution frequency of the SNP rs8099917 among the non-infected individuals and patients complied with Hardy–Weinberg equilibrium. **Table 3** describes the genotypic and allelic frequencies of non-infected individuals and mono- or coinfected patients with chronic hepatitis C.

**Table 3:** Allelic and genotypic frequencies and association analysis – rs8099917.

	Controls (*n* = 199)	Patients (total) (*n* = 230)	Comparison	OR (CI 95%)	*p*
**Genotype**
*TT*	133 (66.8%)	129 (56.1%)	–	–	–
*TG*	62 (31.2%)	94 (40.9%)	GG+TG vs. TT	1.578 (1.044–2.385)	0.029*
*GG*	4 (2.0%)	7 (3.0%)	GG vs. TG+TT	1.530 (0.396–6.314)	0.712
*p*	–	0.033^A^*	–	–	–
**Allele**
* T*	328 (82.4%)	352 (76.5%)	–	–	–
*G*	70 (17.6%)	108 (23.5%)	G vs. T	1.438 (1.013–2.041)	0.041*
*p*	–	0.048^B^*	–	–	–

	**Controls (***n*** = 199)**	**HCV patients (***n*** = 277)**	**Comparison**	**OR (CI 95%)**	*****p*****

**Genotype**
*TT*	133 (66.8%)	96 (54.2%)	–	–	–
*TG*	62 (31.2%)	75 (42.4%)	GG+TG vs. TT	1.700 (1.096–2.640)	0.017*
*GG*	4 (2.0%)	6 (3.4%)	GG vs. TG+TT	1.711 (0.420–7.344)	0.610
*p*	–	0.012^C^*	–	–	–
**Allele**
* T*	328 (82.4%)	267 (75.4%)	–	–	–
*G*	70 (17.6%)	87 (24.6%)	G vs. T	1.527 (1.056–2.209)	0.024*
*p*	–	<0.001^D^*	–	–	–

	**Controls (***n*** = 199)**	**HCV/HIV-1 patients (***n*** = 53)**	**Comparison**	**OR (CI 95%)**	*****p*****

**Genotype**
*TT*	133 (66.8%)	33 (62.3%)	–	–	–
*TG*	62 (31.2%)	19 (35.8%)	GG+TG vs. TT	1.221 (0.621–2.395)	0.645
*GG*	4 (2.0%)	1 (1.9%)	GG vs. TG+TT	0.938 (0.038–9.195)	1.000
*p*	–	0.648^E^	–	–	–
**Allele**
* T*	328 (82.4%)	85 (80.2%)	–	–	–
*G*	70 (17.6%)	21 (19.8%)	G vs. T	1.158 (0.648–2.055)	0.699
*p*	–	0.676^F^	–	–	–

	**HCV patients (***n*** = 177)**	**HCV/HIV-1 patients (***n*** = 53)**	**Comparison**	**OR (CI 95%)**	*****p*****

**Genotype**
*TT*	96 (54.2%)	33 (62.3%)	–	–	–
*TG*	75 (42.4%)	19 (35.8%)	GG+TG vs. TT	0.718 (0.365–1.409)	0.382
*GG*	6 (3.4%)	1 (1.9%)	GG vs. TG+TT	0.548 (0.024–4.762)	0.918
*p*	–	0.327^G^	–	–	–
**Allele**
* T*	267 (75.4%)	85 (80.2%)	–	–	–
*G*	87 (24.6%)	21 (19.8%)	G vs. T	0.758 (0.428–1.334)	0.377
*p*	–	0.357^H^	–	–	–

^A,B^Controls vs. Patients (total); ^C,D^Controls vs. HCV Patients; ^E,F^Controls v*s.* HCV/HIV-1 Patients; ^G,H^HCV Patients vs. HCV/HIV-1 Patients; OR, Odds ratio; CI, Confidence intervals. *Statistical significance.

Similar to rs12979860, significant differences were found in the distribution of frequencies of rs8099917 variants; the *TT* genotype was more frequently observed in non-infected individuals (66.8%, *p* = 0.033) compared with the whole group of patients (56.1%). In addition, the *T* allele was observed more frequently in non-infected individuals (82.4%, *p* = 0.048) compared with the group of mono- and coinfected patients (76.5%). An analysis of the relative risk of this SNP relative to the susceptibility to viral infection identified an OR of 1.530 (*p* = 0.712) for the *GG* genotype alone and an OR of 1.578 (*p* = 0.029) for the genotypes containing at least one allele *G* (*GG* and *TG*) in the patients with chronic hepatitis C (mono- and coinfected patients together). Similar result was obtained in the analysis of the relative risk of the *G* allele (OR = 1.438, *p* = 0.041).

Significant differences were also observed in the distribution of genotypes and alleles in non-infected individuals compared with the group of mono-infected patients (**Table 3**). As before, the *TT* genotype was observed significantly more often in non-infected individuals (66.8%, *p* = 0.012), as well as *T* allele (82.4%, *p* < 0.001), compared with the group of mono-infected patients (54.2 and 75.4%, respectively). However, these differences were not observed when the non-infected individuals were compared with the coinfected patients. An analysis of the relative risk of this SNP relative to the susceptibility to viral infection in the mono-infected patients indicated an OR of 1.711 (*p* = 0.610) for the *GG* genotype alone, an OR of 1.700 (*p* = 0.017) for the genotypes containing at least one allele *G* (*GG* and *TG*), and an OR of 1.527 (*p* = 0.024) for the *G* allele. In the coinfected patients, the ORs of the *GG* genotype, the *GG*+*TG* genotypes, and the *G* allele were 0.938 (*p* = 1.000), 1.221 (*p* = 0.645), and 1.158 (*p* = 0.699), respectively, without statistical significance.

As with rs12979860, we observed no significant differences in the distribution of the *TT* genotype (54.2% vs. 62.3%, *p* = 0.327) or the *T* allele (75.4% vs. 80.2%, *p* = 0.357) between the mono-infected and coinfected patients, respectively. Neither risk factor analysis revealed significant differences with respect to either the genotypes (*GG* vs. *TG*+*TT*, OR = 0.548, *p* = 0.918; *GG*+*TG* vs. *TT*, OR = 0.718, *p* = 0.382) or alleles (OR = 0.758, *p* = 0.377).

## DISCUSSION

According to the World Health Organization [WHO] (2014), 350.000–500.000 people die every year from hepatitis C-related illnesses. The mechanisms underlying the high HCV infection persistence rate have not yet been fully elucidated. The existence of *quasispecies* and the wide mutation capability favor the constant escape of the virus from the host immune response (Strauss, 2001). Some virus- and host-related factors have been associated with the persistence of HCV infection in affected individuals, including the serum levels of HCV RNA, ethnicity, coinfection with HIV, human leukocyte antigens (HLA) alleles, alcohol consumption, age at infection, and gender (Hofer et al., 2003; Seeff and Hoofnagle, 2003; Yee, 2004; Hong et al., 2005; Thomas et al., 2009; Rao et al., 2011). However, data on the genetic host factors involved in the spontaneous clearance of infection have been scarce until recently (García et al., 2001).

*IL28B* gene encodes interferon-λ3, which belongs to an interferon family characterized by powerful antiviral activity. This cytokine is able to activate the cell signaling pathways that control viral replication, e.g., the interferon-stimulated genes (ISG; Balagopal et al., 2010). Recent studies have demonstrated the impact of so-called favorable genotypes and alleles (*CC* and *TT* genotypes, *C* and *T* alleles of polymorphisms rs8099917 and rs12979860, respectively) on the response to the standard treatment for chronic hepatitis C, as well as their relationship with spontaneous HCV clearance in mono- and HIV coinfected individuals (Ge et al., 2009; Suppiah et al., 2009; Tanaka et al., 2009; Thomas et al., 2009; Aparicio et al., 2010; Rállon et al., 2010; Rauch et al., 2010).

One of the questions addressed by the present study concerns the effect of the SNPs rs12979860 and rs8099917 on the host resistance/susceptibility to HCV infection, which remains poorly understood. García et al. (2001) raised the hypothesis that *IL28B*-related polymorphisms might modulate, at least partially, the ISGs and thus alter the natural history of HCV infection.

The study of Sarrazin et al. (2011), conducted in Germany, identified significant differences in the distribution of SNP rs12979860 genotypes among the patients infected by HCV genotype 1 compared with non-infected individuals. The frequency of the *CC* genotype was 49% in the group of non-infected individuals (*n* = 200) vs. 33.9% in the group of patients (*n* = 378, *p* < 0.001). The authors suggested that the unfavorable rs12979860 genotype (*TT*) acts by predisposing the individuals to chronic infection.

The study by Shi et al. (2012) in a Chinese population demonstrated that the expression of the *IL28B* gene was significantly reduced in a group of patients with chronic hepatitis C (*n* = 529), compared with individuals who exhibited spontaneous viral clearance (*n* = 196, *p* = 0.003) and with non-infected individuals (*n* = 171, *p* = 0.04). The authors suggested that the SNP rs12979860 variants might exert a direct influence on the expression of *IL28B* and thus on the production of interferon-λ3. Similar results were observed in the German study conducted by Langhans et al. (2011), who noted that the levels of the *IL28A/B* genes were significantly higher among the carriers of the *C* allele compared with the homozygous *TT* individuals (IL28A/B, *p* = 0.009).

In the study by Beinhardt et al. (2012), conducted in Austria, the rs12979860 *CC* genotype was observed more frequently among patients with acute hepatitis C (*n* = 120) compared to the controls (*n* = 96; 62.5% vs. 39.6%, *p* < 0.001) and was also observed more frequently among patients who exhibited spontaneous viral clearance compared to those with viral persistence (74.6% vs. 51.7%, *p* = 0.021).

In the meta-analysis by Aalaei-Andabili et al. (2014), it was found that rs12979860 *CC* genotype and *C* allele prevalences among healthy subjects were similar to their distributions in genotypes 2 and 3 HCV infected patients (favorable HCV genotypes) in caucasian ethnicity, but they significantly differed from the distributions in genotypes 1 and 4 HCV infected patients. It seems that genotypes 1 and 4 HCV infected patients with a higher rate of rs12979860 *CC* genotype and *C* allele could better eradicate HCV RNA in the acute phase of HCV infection, versus genotypes 2 and 3. This ability leads to a lower rate of rs12979860 *CC* prevalence in the chronic phase of HCV infected patients, compared to genotypes 2 and 3.

The study by Lunge et al. (2012) sought to assess the correlation between the SNP rs12979860 and the spontaneous clearance of HCV in a population of Brazilian HIV coinfected patients. The genotype distribution differed significantly in the group with spontaneous viral clearance (*CC*: 52.9, *CT*: 32.4, and *TT*: 14.7%) compared to the patients with chronic infection (*CC*: 28.8, *CT*: 60.6, and *TT*: 10.6%) (*p* < 0.05). The *CT* and *TT* genotypes correlated with an almost threefold increase of the odds of developing chronic infection compared to the *CC* genotype (OR = 2.78, *p* = 0.011).

In the present study, the results of the relative risk analysis of the rs12979860 genotypes and alleles relative to the susceptibility to HCV infection indicate that the *TT*+*CT* genotypes and *T* allele exhibit a significant association with the suggested hypothesis. Curiously, the subgroup of coinfected patients exhibited greater risk (OR = 3.000) when heterozygotes were included in the analysis compared with the individuals with the *TT* genotype alone (OR = 1.583), which was further supported by the lack of statistical significance of the *TT* homozygotes.

Some authors have discussed the distribution and impact of the rs8099917 variants in patients and controls. The Brazilian study conducted by Ramos et al. (2012) with patients mono-infected by HCV genotype 1 reported 48% patients with the *TT* genotype, 19% with the *TG* genotype, and 33% with the *GG* genotype, which differs from the genotypic frequencies observed in the present study – 54.2, 42.4 and 3.4%, respectively. Tanaka et al. (2009) conducted a genome-wide association study (GWAS) in 154 Japanese patients infected by HCV genotype 1 and observed a correlation between the *G* allele and the low expression of the *IL28B* gene. The study by Aparicio et al. (2010) with 160 HCV/HIV coinfected patients in Spain observed a high frequency of the *G* allele in patients infected by HCV genotypes 1 (40%) and 4 (42%).

The study by Vassilev et al. (2012) in Bulgaria sought to assess the allelic frequency of rs8099917 in the overall population (*n* = 79) and in a group of patients with chronic liver diseases (*n* = 176). The authors observed that the favorable allele *(T)* occurred more frequently in the overall population (*p* = 0.0071). Beinhardt et al. (2012) observed the rs8099917 *TT* genotype more frequently in patients with acute hepatitis C compared with the controls (83.3% vs. 69.8%, *p* = 0.018); the *TT* genotype was also observed more frequently among the individuals who exhibited spontaneous HCV clearance compared with those in whom the virus persisted (91.5% vs. 75.0%, *p* = 0.025).

In the present study, an analysis of the relative risk of the rs8099917 genotypes demonstrated that the *GG*+*TG* genotypes and the *G* allele exhibited a positive correlation with chronic infection by HCV in the patient population, however, none of these correlations were observed in the group of coinfected patients alone.

In both evaluated polymorphisms, the absence of an observed effect in the group of HIV-coinfected patients does not imply necessarily an absence of association. The small number of patients sampled in this group might be a contributing factor for this result. The absence of a significant difference in the polymorphic distribution of both genotypes and alleles between the mono- and the coinfected patients, in addition to the lack of significance in the relative risk analysis, suggests that the results of the present study apply to chronic HCV carriers and HIV coinfected patients. A possible association of *IL28B* polymorphisms with the patient’s condition on recruitment or clinical outcome was not investigated since it did not belong to the study objectives.

Martin et al. (2010) and Nattermann et al. (2011) did not observe any correlation between the SNP rs12979860 and HIV mono-infected patients. There was no significant difference in the susceptibility to HIV infection, progression of infection, or the clinical and laboratory parameters, such as serum viral RNA levels and CD4^+^ cell count. Therefore, HIV appears to be associated with SNPs near the *IL28B* gene only when there is coinfection by HCV. The present study was not able to elucidate whether HIV plays any role in the establishment of HCV infection.

Linkage disequilibrium between both SNPs revealed they were highly associated in all groups of the study, suggesting a non-random association that comprise the two main polymorphisms near the *IL28B* gene. Similar results were reported by Suppiah et al. (2009), Tanaka et al. (2009), Rauch et al. (2010), and Cavalcante et al. (2012).

*IL28B* gene polymorphisms are known to be the most important predictors for SVR to Peg-IFN/ribavirin therapy in genotype 1/4 and, on a smaller scale, in genotype 2/3 infected patients for triple-therapies based on BOC/TVR in treatment-naїve patients (Ge et al., 2009; Rauch et al., 2010; Jacobson et al., 2011; Poordad et al., 2011; Fischer et al., 2012; Jiménez-Sousa et al., 2013; Pol et al., 2013). The knowledge about variants of *IL28B* gene polymorphisms is useful for decision-making therapy, providing individualized treatment options (Matsuura et al., 2014). For example, patients who harbor the favorable genotype may receive the usual treatment, while patients who have an unfavorable genotype and/or are at higher risk of disease evolution shall be treated with triple-therapy (Imazeki et al., 2010; Petta and Craxí, 2013). Moreover, patients with favorable genotype are more likely to achieve SVR, possibly shortening the treatment time. Shorter treatment schemes result in lower costs and also encourage patients to finish the treatment (Georgel et al., 2010).

In conclusion, the frequencies of the rs12979860 *C* allele and *CC* genotype and the rs8099917 *T* allele and *TT* genotype were significantly higher in the group of non-infected individuals compared with mono- and HIV-1 coinfected patients with chronic HCV infection. The rs12979860 *TT* genotype and *T* allele behaved as significant risk factors for HCV infection in the mono- and coinfected patients, whereas the rs8099917 *GG* genotype and *G* allele represented risk factors for the mono-infected patients in particular. The present study was unable to clarify whether rs12979860 and rs8099917 variants exert a significant influence on the establishment of HCV infection in individuals also infected with HIV. Based on our results, we suggest that SNPs near the *IL28B* gene exert a significant influence on the establishment of HCV infection, contributing to a better understanding of the susceptibility to and the natural history of hepatitis C.

## Conflict of Interest Statement

The authors declare that the research was conducted in the absence of any commercial or financial relationships that could be construed as a potential conflict of interest.
